# A NMR-Based Metabonomics Approach to Determine Protective Effect of a Combination of Multiple Components Derived from Naodesheng on Ischemic Stroke Rats

**DOI:** 10.3390/molecules24091831

**Published:** 2019-05-13

**Authors:** Lan Luo, Jiazhen Kang, Qiong He, Yue Qi, Xingyu Chen, Shumei Wang, Shengwang Liang

**Affiliations:** 1 School of Traditional Chinese Medicine, Guangdong Pharmaceutical University, Guangzhou 510006, China; luolan1105@126.com (L.L.); KangJZI@163.com (J.K.); m15975633308@163.com (Q.H.); qiyue9339@163.com (Y.Q.); witwick@163.com (X.C.); shmwang@sina.com (S.W.); 2 Key Laboratory of Digital Quality Evaluation of Chinese Materia Medica of State Administrationof TCM, Guangdong Pharmaceutical University, Guangzhou 510006, China; 3 Engineering & Technology Research Center for Chinese Materia Medica Quality of the Universities of Guangdong Province, Guangdong Pharmaceutical University, Guangzhou 510006, China

**Keywords:** NMR-based metabonomics, biochemical assessment, Naodesheng, a combination of multiple components, ischemic stroke, middle cerebral artery occlusion

## Abstract

Naodesheng (NDS) is a widely used traditional Chinese medicine (TCM) prescription for the treatment of ischemic stroke. A combination of 10 components is derived from NDS. They are: Notoginsenoside R1, ginsenoside Rg1, ginsenoside b1, ginsenoside Rd, hydroxysafflor yellow A, senkyunolide I, puerarin, daidzein, vitexin, and ferulic acid. This study aimed to investigate the protective effect of the ten-component combination derived from NDS (TCNDS) on ischemic stroke rats with a middle cerebral artery occlusion (MCAO) model by integrating an NMR-based metabonomics approach with biochemical assessment. Our results showed that TCNDS could improve neurobehavioral function, decrease the cerebral infarct area, and ameliorate pathological features in MCAO model rats. In addition, TCNDS was found to decrease plasma lactate dehydrogenase (LDH) and malondialdehyde (MDA) production and increase plasma superoxide dismutase (SOD) production. Furthermore, ^1^H-NMR metabonomic analysis indicated that TCNDS could regulate the disturbed metabolites in the plasma, urine, and brain tissue of MCAO rats, and the possible mechanisms were involved oxidative stress, energy metabolism, lipid metabolism, amino acid metabolism, and inflammation. Correlation analysis were then performed to further confirm the metabolites involved in oxidative stress. Correlation analysis showed that six plasma metabolites had high correlations with plasma LDH, MDA, and SOD. This study provides evidence that an NMR-based metabonomics approach integrated with biochemical assessment can help to better understand the underlying mechanisms as well as the holistic effect of multiple compounds from TCM.

## 1. Introduction

Stroke remains a leading cause of mortality and disability worldwide. The pathophysiology of acute ischemic stroke is very intricate, involving excitotoxicity damage, inflammatory mediators, oxidative stress, and apoptosis [1]. At present, safe and effective treatment has been limited. There is thus an urgent need to develop agents with high efficacy and low side effects.

Naodesheng (NDS) is a widely used traditional Chinese medicine (TCM) prescription in the clinic treatment of cerebral infarction with good therapeutic effects [2,3]. NDS is composed of five traditional Chinese medicinal herbs, namely Radix Notoginseng, Rhizoma Chuanxiong, Flos Carthami, Fructus Crataegi, and Radix Puerariae. NDS was reported to significantly decrease the infarction area and alleviate neurological function deficits in focal cerebral ischemia rats [4]. However, the effective components of NDS, as well as the mechanisms underlying the effects, are still unclear.

Metabolomics, a powerful tool for disease fingerprinting and biomarker discovery, can systematically obtain the qualitative and quantitative information of endogenous metabolites [5]. Metabolite profiles in biological samples can be measured by using several techniques, such as nuclear magnetic resonance (NMR), gas chromatography-mass spectrometry (GC-MS), and liquid chromatography-mass spectrometry (LC-MS). NMR metabolomics, one of the most preferred methods, can detect a wide variety of compound families and can thus be particularly suitable for untargeted analysis [6]. In the past few years, an NMR-based metabonomics approach has been proved to be successfully applied for biological samples, such as plasma, serum, urine, brain tissue, and cerebrospinal fluid [7,8,9,10,11].

In our previous study, NDS was confirmed to exert antithrombotic and neuroprotective effects [12,13]. In addition, the contents of ten components in NDS extract were determined by the LC-MS method. These included notoginsenoside R1 (NGR1), ginsenoside Rg1 (GSRg1), ginsenoside b1 (GSb1), ginsenoside Rd (GSRd), hydroxysafflor yellow A (HSYA), senkyunolide I (SI), puerarin (PA), daidzein (DZ), vitexin (VT), and ferulic acid (FA) [14]. Furthermore, we developed and validated a simple and reliable LC-MS/MS method for the simultaneous quantification of these components in rat serum, and the method was successfully applied to a pharmacokinetic study of these components in rat serum after oral administration of NDS [15].

Based on our previous studies, this study was designed to investigate the protective effect of the ten-component combination derived from NDS (TCNDS) on ischemic stroke rats with the middle cerebral artery occlusion (MCAO) model by integrating an NMR-based metabonomics approach with biochemical assessment, including plasma lactate dehydrogenase (LDH), malondialdehyde (MDA), and superoxide dismutase (SOD). 

## 2. Results

### 2.1. Neurobehavioral Dysfunction Evaluation of TCNDS on MCAO Rats

Scores on neurobehavioral performance are shown in Table 1. The higher the score, the more serious the neurological impairment. It was found that the scores in nimodipine, low dosage of TCNDS (L-TCNDS), and high dosage of TCNDS (H-TCNDS) groups were significantly lower than that in the model group, indicating that nimodipine and TCNDS could significantly alleviate the neurological symptoms.

### 2.2. Effect of TCNDS on Cerebral Infarct Area and Pathological Changes in MACO Rats 

**Figure 1:**
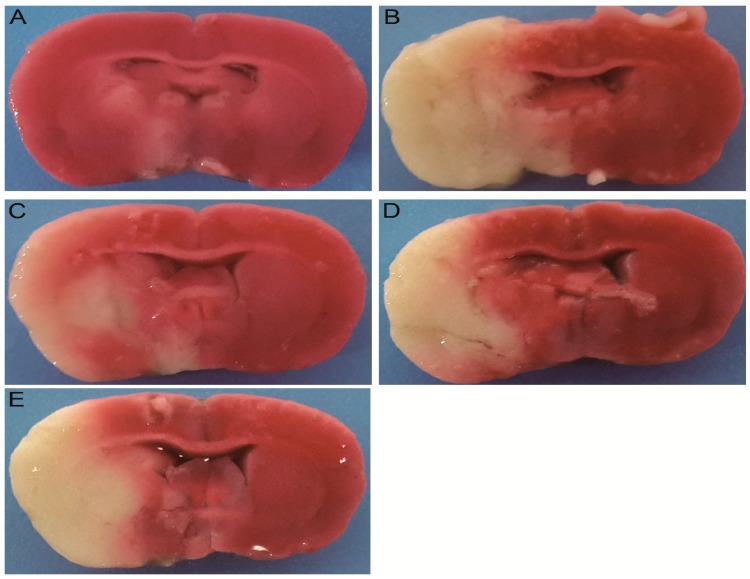
Triphenyltetrazolium chloride (TTC) staining results of brain tissue: The control group (**A**), model group (**B**), nimodipine group (**C**), high dosage of the ten-component combination derived from Naodesheng (NDS) (TCNDS) (H-TCNDS) group (**D**), and low dosage of TCNDS (L-TCNDS) group (**E**). H-TCNDS meaned high dosage of TCNDS (316.34 mg·kg^−1^·d^−1^), and L-TCNDS meaned low dosage of TCNDS (158.17 mg·kg^−1^·d^−1^).

**Figure 2:**
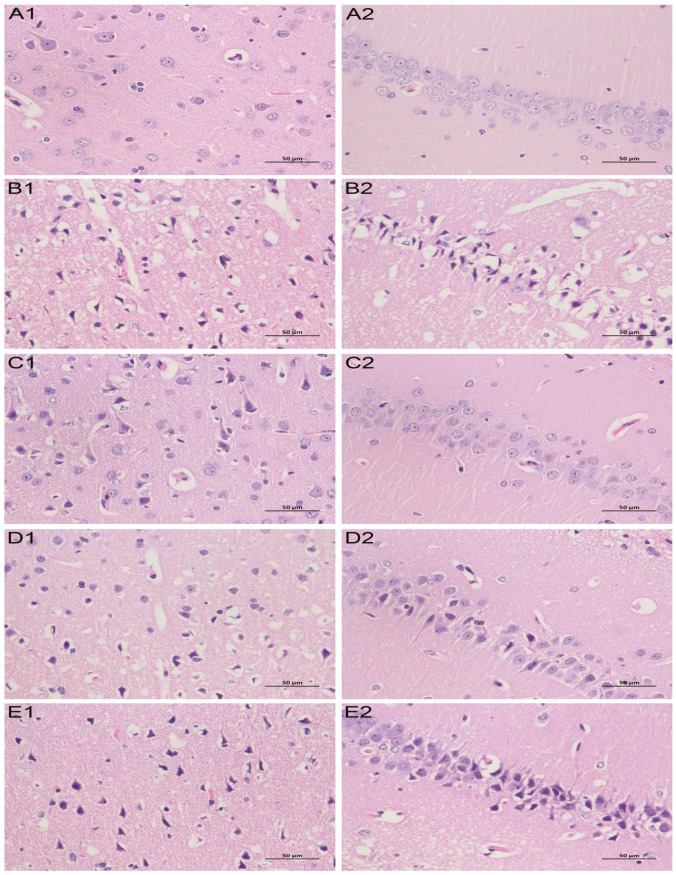
Hematoxylin-eosin (HE) staining results of brain tissue (×400): Nerve cells in the cerebral cortex for the control group (**A1**), model group (**B1**), nimodipine group (**C1**), H-TCNDS group (**D1**), and L-TCNDS group (**E1**). Nerve cells in the CA1 region of hippocampus for the control group (**A2**), model group (**B2**), nimodipine group (**C2**), H-TCNDS group (**D2**), and L-TCNDS group (**E2**). H-TCNDS meaned high dosage of TCNDS (316.34 mg·kg^−1^·d^−1^), and L-TCNDS meaned low dosage of TCNDS (158.17 mg·kg^−1^·d^−1^).

Triphenyltetrazolium chloride (TTC) staining results indicated a significant increase in the cerebral infarct area of the model group compared to the control group, as seen in Figure 1 and Table 1. However, a significant reduction in the cerebral infarct area was seen in nimodipine and TCNDS groups compared to the model group. Moreover, hematoxylin-eosin (HE) staining results showed most apoptosis of nerve cells in the cerebral cortex and hippocampal CA1 region from the model group compared to the control group, as seen in Figure 2. There appeared to be severe swelling, disordered arrangement, and karyopyknosis in the nerve cells of the model group. However, the abnormal cell morphology induced by a cerebral ischemia/reperfusion (I/R) injury was improved by the nimodipine and TCNDS groups. These findings indicated that L-TCNDS and H-TCNDS could exert therapeutic effect on MACO rats, consistent with neurobehavioral dysfunction evaluation.

### 2.3. Effect of TCNDS on Plasma LDH, MDA and SOD Levels in MCAO Rats

As shown in Table 2, compared to the control group, a significant elevation in the plasma levels of LDH and MDA, together with a significant reduction in plasma SOD level, was seen in the model group. However, nimodipine and TCNDS groups could reverse effectively the abnormal levels of LDH, MDA, and SOD in plasma, which were induced by a cerebral ischemia/reperfusion (I/R) injury.

### 2.4. Effect of TCNDS on Metabolic Profiles of MCAO Rats by ^1^H-NMR-Based Metabonomics

**Figure 3:**
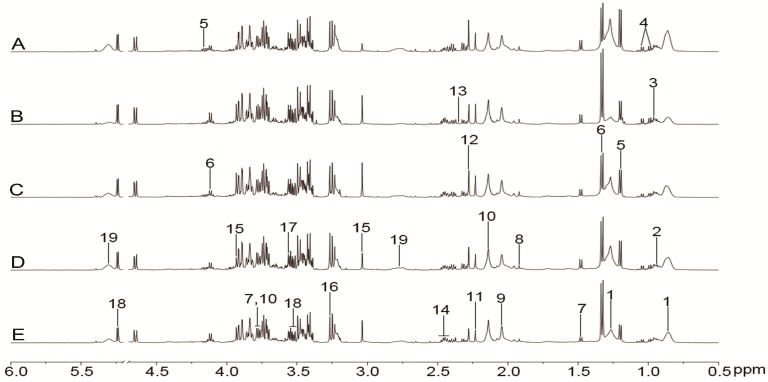
Representative ^1^H-NMR spectra (0.5–6.0 ppm) of plasma for the control (**A**), model (**B**), nimodipine (**C**), H-TCNDS (**D**), and L-TCNDS (**E**) groups. Key metabolites: 1. VLDL/LDL; 2. isoleucine; 3. leucine; 4. valine; 5. 3-hydroxybutyrate; 6. lactate; 7. alanine; 8. acetate; 9. *N*-acetyl glycoprotein; 10. methionine; 11. acetone; 12. acetoacetate; 13. Pyruvate; 14. glutamine; 15. creatine/phosphocreatine; 16. trimethylamine-*N*-oxide /betaine; 17. glycine; 18. α-glucose; 19. poly unsaturated fatty acid. H-TCNDS meaned high dosage of TCNDS (316.34 mg·kg^−1^·d^−1^), and L-TCNDS meaned low dosage of TCNDS (158.17 mg·kg^−1^·d^−1^).

**Figure 4:**
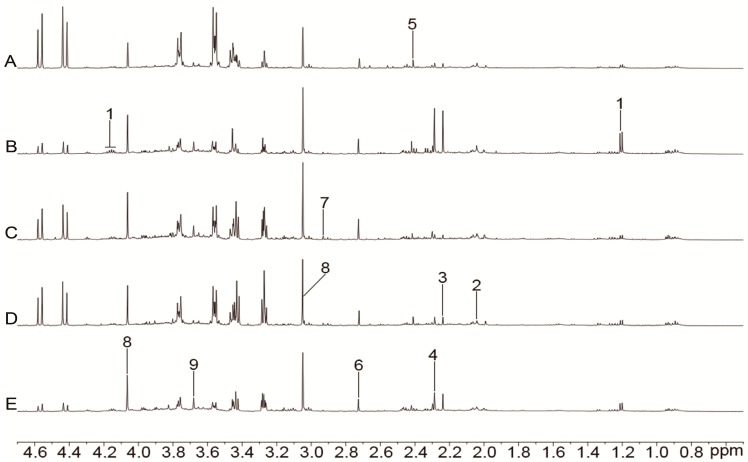
Representative ^1^H-NMR spectra (0.5–4.7 ppm) of urine for the control (**A**), model (**B**), nimodipine (**C**), H-TCNDS (**D**), and L-TCNDS (**E**) groups. Key metabolites: 1. 3-hydroxybutyrate; 2. *N*-acetyl glycoprotein; 3. acetone; 4. acetoacetate; 5. succinate; 6. dimethylamine; 7. dimethylglycine; 8. creatinine; 9. phenylacetyl-glycine. H-TCNDS meaned high dosage of TCNDS (316.34 mg·kg^−1^·d^−1^), and L-TCNDS meaned low dosage of TCNDS (158.17 mg·kg^−1^·d^−1^).

**Figure 5:**
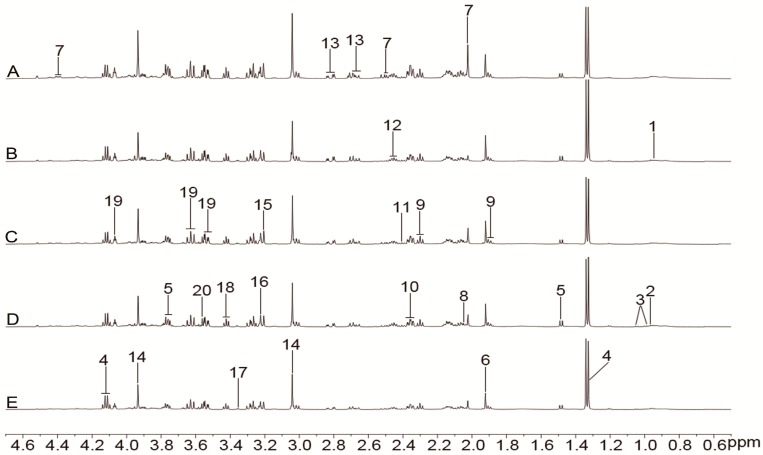
Representative ^1^H-NMR spectra (0.5–4.7 ppm) of brain tissue for the control (**A**), model (**B**), nimodipine (**C**), H-TCNDS (**D**), and L-TCNDS (**E**) groups. Key metabolites: 1. isoleucine; 2. leucine; 3. valine; 4. lactate; 5. alanine; 6. acetate; 7. *N*-acetyl-aspartate; 8. *N*-acetylaspartylglutamate; 9. γ-aminobutyric acid; 10. glutamate; 11. succinate; 12. glutamine; 13. aspartate; 14. creatine/phosphocreatine; 15. choline; 16. phosphorylcholine/glycerophosphocholine; 17. scyllo-inositol; 18. taurine; 19. myo-inositol; 20. glycine. H-TCNDS meaned high dosage of TCNDS (316.34 mg·kg^−1^·d^−1^), and L-TCNDS meaned low dosage of TCNDS (158.17 mg·kg^−1^·d^−1^).

**Figure 6:**
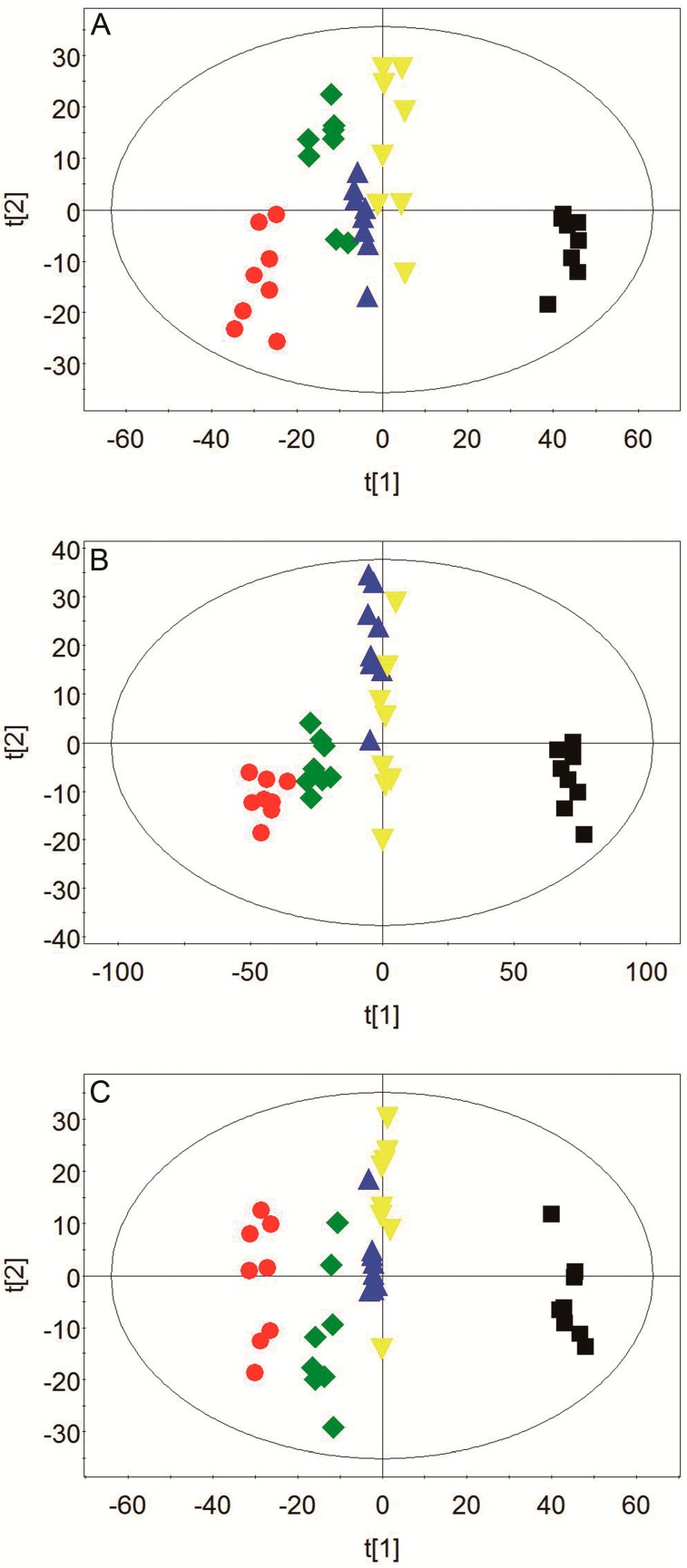
Scores plots of partial least squares discriminant analysis (PLS-DA) for plasma (**A**), urine (**B**), and brain tissue (**C**). ● Control group; ■ Model group; ◆ Nimodipine group; ▲ H-TCNDS group; ▼ L-TCNDS group. H-TCNDS meaned high dosage of TCNDS (316.34 mg·kg^−1^·d^−1^), and L-TCNDS meaned low dosage of TCNDS (158.17 mg·kg^−1^·d^−1^).

The representative ^1^H-NMR spectra of plasma, urine, and brain tissue are shown in Figure 3, Figure 4 and Figure 5 with metabolites labeled. Major endogenous metabolites were assigned according to the previously reported data [16,17,18] and two-dimensional (2D) NMR (Figures S1–S6). ^1^H-NMR data of these metabolites in rat plasma, urine, and brain tissue are listed in Tables S1–S3. Principal component analysis (PCA) was firstly performed for all groups in plasma, urine, and brain tissue. All the PCA results (Figure S7) showed that none of the spots was located outside of the confidence interval. Partial least squares discriminant analysis (PLS-DA) was then used to enlarge the distinction between groups. As shown in Figure 6, it was found that the nimodipine and TCNDS groups were clearly separated from the model group, and they were drawn close to the control group, indicating that TCNDS relieved the metabolic disorders in plasma, urine, and brain tissue and thereby exerted protective effect on MCAO rats in agreement with neurobehavioral and histopathological assessments.

**Figure 7:**
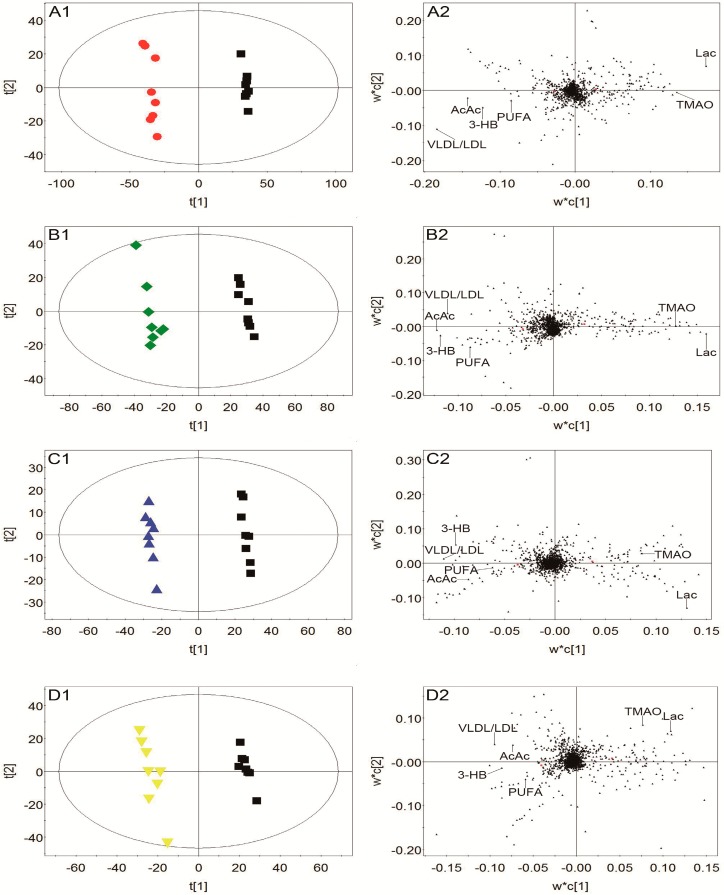
Score plots and loading plots of PLS-DA from plasma: Score plot (**A1**) and loading plot (**A2**) of the model and control groups, score plot (**B1**) and loading plot (**B2**) of the model and nimodipine groups, score plot (**C1**) and loading plot (**C2**) of the model and H-TCNDS groups, and score plot (**D1**) and loading plot (**D2**) of the model and L-TCNDS groups. ● Control group; ■ Model group; ◆ Nimodipine group; ▲ H-TCNDS group; ▼ L-TCNDS group. H-TCNDS meaned high dosage of TCNDS (316.34 mg·kg^−1^·d^−1^), and L-TCNDS meaned low dosage of TCNDS (158.17 mg·kg^−1^·d^−1^).

**Figure 8:**
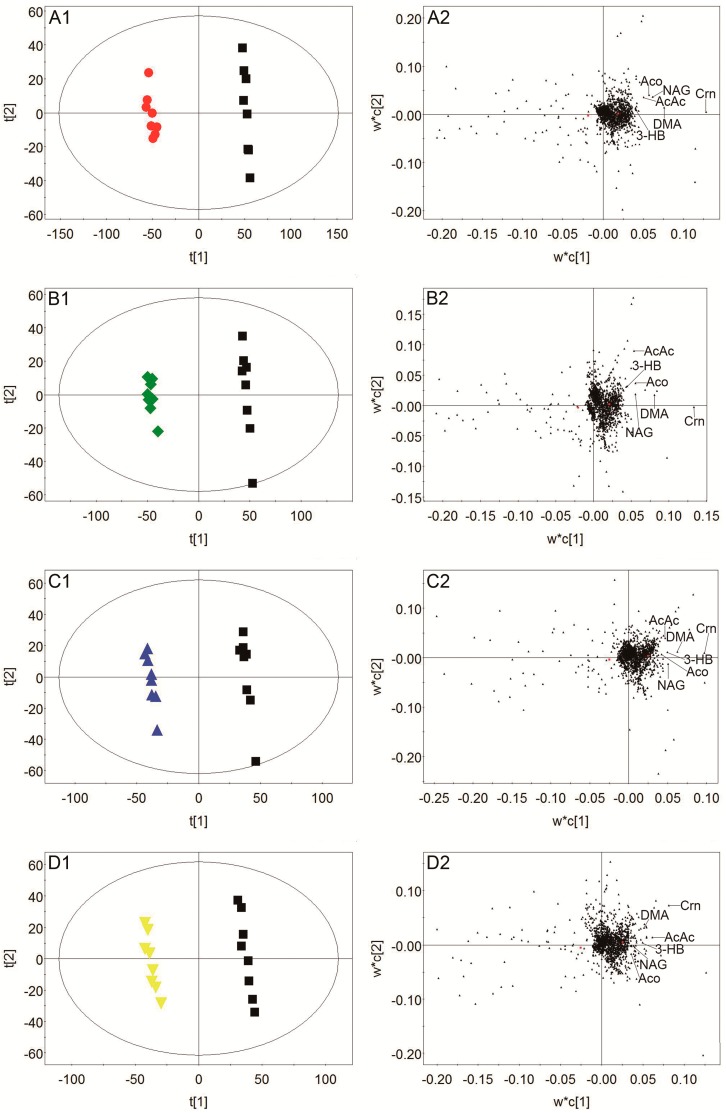
Score plots and loading plots of PLS-DA from urine: Score plot (**A1**) and loading plot (**A2**) of the model and control groups, score plot (**B1**) and loading plot (**B2**) of the model and nimodipine groups, score plot (**C1**) and loading plot (**C2**) of the model and H-TCNDS groups, and score plot (**D1**) and loading plot (**D2**) of the model and L-TCNDS groups. ● Control group; ■ Model group; ◆ Nimodipine group; ▲ H-TCNDS group; ▼ L-TCNDS group. H-TCNDS meaned high dosage of TCNDS (316.34 mg·kg^−1^·d^−1^), and L-TCNDS meaned low dosage of TCNDS (158.17 mg·kg^−1^·d^−1^).

**Figure 9:**
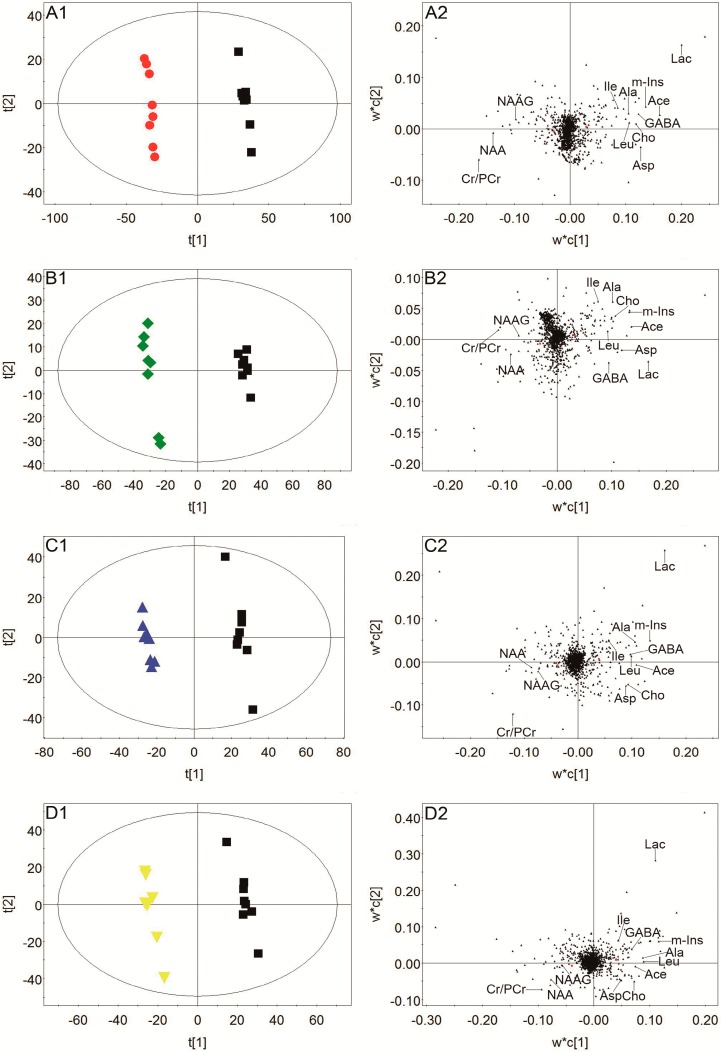
Score plots and loading plots of PLS-DA from brain tissue: Score plot (**A1**) and loading plot (**A2**) of the model and control groups, score plot (**B1**) and loading plot (**B2**) of the model and nimodipine groups, score plot (**C1**) and loading plot (**C2**) of the model and H-TCNDS groups, and score plot (**D1**) and loading plot (**D2**) of the model and L-TCNDS groups. ● Control group; ■ Model group; ◆ Nimodipine group; ▲ H-TCNDS group; ▼ L-TCNDS group. H-TCNDS meaned high dosage of TCNDS (316.34 mg·kg^−1^·d^−1^), and L-TCNDS meaned low dosage of TCNDS (158.17 mg·kg^−1^·d^−1^).

To discover the potential biomarkers associated with ischemic stroke, PLS-DA models were constructed between the model group and control group. A clear classification between the model group and control group was observed in plasma, urine, and brain tissue, as seen in Figure 7A1, Figure 8A1, and Figure 9A1, respectively, indicating that metabolic profiles of plasma, urine, and brain tissue in MCAO rats were significantly changed after a cerebral I/R injury. Parameters of these PLS-DA models were as follows: R^2^Y = 0.997, Q^2^ = 0.986 for plasma; R^2^Y = 0.998, Q^2^ = 0.986 for urine; and R^2^Y = 0.999, Q^2^ = 0.983 for brain tissue. Moreover, 200-interation permutation tests were performed, which demonstrated that these PLS-DA models were stable and good to fitness, as shown in Figures S8A, S9A, and S10A. Discriminatory metabolites between the model group and control group in plasma, urine, and brain tissue were selected out by using variable importance for the projections (VIP) values (VIP > 1) and *p* < 0.05. From the loading plot (Figure 7A2), it was found that the lactate (Lac) and trimethylamine-*N*-oxide/betaine (TMAO/Bet) levels in plasma were increased, but the VLDL/LDL, 3-hydroxybutyrate (3-HB), acetoacetate (AcAc), and poly unsaturated fatty acid (PUFA) levels in plasma were decreased in model rats compared to normal control rats. In addition, Figure 8A2 shows that six discriminatory metabolites levels in urine were elevated in model rats compared to normal control rats, including creatinine (Crn), AcAc, acetone (Aco), *N*-acetyl glycoprotein (NAG), dimethylamine (DMA), and 3-HB. In addition, Figure 9A2 shows that concentrations of Lac, acetate (Ace), myo-inositol (m-Ins), γ-aminobutyric acid (GABA), aspartate (Asp), alanine (Ala), leucine (Leu), isoleucine (Ile), and choline (Cho) in brain tissue were elevated, but concentrations of creatine/phosphocreatine (Cr/PCr), *N*-acetyl-aspartate (NAA), and *N*-acetylaspartylglutamate (NAAG) in brain tissue were reduced in model rats compared to normal control rats. These potential biomarkers in plasma, urine, and brain tissue associated with ischemic stroke are listed in Table 3.

To elucidate the regulation of H-TCNDS and L-TCNDS on metabolic profiles of MCAO rats, PLS-DA models from plasma, urine, and brain tissue were established between the model and TCNDS groups. These model parameters are shown in Table S4. Additionally, the results of 200-interation permutation tests from plasma, urine, and brain tissue are shown in Figures S8, S9, and S10, respectively. From the loading plot (Figure 7C2,D2), it was found that H-TCNDS and L-TCNDS could regulate the abnormal concentrations of VLDL/LDL, 3-HB, AcAc, Lac, TMAO/Bet, and PUFA in plasma towards normal. Moreover, they could decrease the elevated urine concentrations of Crn, AcAc, Aco, NAG, DMA, and 3-HB induced by a cerebral I/R injury, as shown in Figure 8C2,D2. In addition, they could reverse the disturbed metabolites in brain tissue, including Lac, Cr/PCr, Ace, NAA, m-Ins, GABA, Asp, Leu, Ala, Ile, Cho, and NAAG, as seen in Figure 9C2,D2.

### 2.5. Correlation between Biochemical Factors and Discriminatory Metabolites in Plasma.

In order to further confirm the discriminatory metabolites involved in oxidative stress, Pearson correlation analysis was used between discriminatory metabolites and biochemical factors for the control group vs the model group in plasma. A correlation coefficient ≥ 0.7 expressed high positive correlation, while a correlation coefficient ≤ −0.7 expressed high negative correlation [19]. Six plasma metabolites had high correlations with plasma LDH, MDA, and SOD, as seen in Table 4.

## 3. Discussion

In this study, we established a MCAO rat model to imitate human ischemic stroke. Using a MCAO rat model, we indicated the protective effect of TCNDS against a cerebral I/R injury in rat, as evidenced by improved neurobehavioral function, decreased cerebral infarct area, and ameliorated pathological features.

The findings of biochemical assessment showed that a cerebral I/R injury could lead to an increase in plasma LDH level, consistent with the previous study [20]. LDH, an indirect marker of hypoxia, was measured to evaluate cerebral ischemia damage. Our results indicated that TCNDS could reduce plasma LDH level in MCAO rats. Additionally, it was found that a cerebral I/R injury could induce an elevation in plasma MDA level, as well as a reduction in plasma SOD level, in agreement with the previous study [21]. MDA, an index of oxidative damage, was measured to estimate the end product of lipid peroxidation [22]. It was reported that reduction in the SOD level during brain ischemia could increase neural cell death and brain injury [23]. Our results indicated that TCNDS could regulate the abnormal levels of SOD and MDA in plasma, suggesting its protective effect against oxidative stress.

Oxidative stress was thought to play an important role in the pathophysiology of a cerebral I/R injury, leading to lipid peroxidation and protein oxidization [24,25,26,27]. In this study, reduced plasma levels of PUFA and VLDL/LDL, together with high correlations with plasma MDA and SOD, were seen in MCAO rats, associated with fatty acids and lipid protein oxidization. Oxidized LDL was thought to be a high risk indicator in cardiovascular diseases [28]. Our results indicated the regulation of TCNDS on the abnormal levels of plasma PUFA and VLDL/LDL. In addition, increased brain levels of Leu and Ile were observed in MCAO rats, possibly due to protein oxidation and degradation. Our results showed the regulation of TCNDS on the abnormal levels of brain Leu and Ile. In addition, elevated brain levels of Cho and m-Ins were observed in MCAO rats, possibly related to cell membrane lipid oxidation. TMAO was generated from Cho and then decomposed into DMA. Accordingly, increased levels of plasma TMAO/Bet and urine DMA were seen in MCAO rats. Furthermore, high correlations between plasma TMAO/Bet with plasma MDA and SOD were found. Our results indicated that TCNDS could regulate the disturbed metabolites, including brain Cho, brain m-Ins, plasma TMAO/Bet, and urine DMA.

Energy metabolism dysfunction was reported to be induced by a cerebral I/R injury [29]. In this study, a great number of plasma Lac were produced, together with the high positive correlation with plasma LDH after a cerebral I/R injury, possibly due to depressed aerobic metabolism. Our results showed that TCNDS could reduce Lac levels in plasma and brain tissue, possibly associated with improvement of energy supply pattern. Additionally, TCNDS could decrease the increased level of brain Ala in MCAO rats, which might be a result of improvement of the glucose-alanine cycle [16].

The ketone bodies, as an alternative fuel source, are produced in the liver in a condition of decreased carbohydrate availability, such as AcAc, 3-HB and Aco. In this study, we found that the urine concentrations of AcAc, 3-HB, and Aco were elevated in MCAO rats, probably related to a stimulation of ketogenesis by a cerebral I/R injury. Additionally, significantly decreased concentrations of plasma AcAc and 3-HB were seen in MCAO rats, in agreement with the previous report [10]. Our results indicated that TCNDS could regulate the abnormal concentrations of AcAc, 3-HB, and Aco, associated with improved energy supply.

Cr, an important role in brain energy metabolism, serves in the regeneration of adenosine triphosphate (ATP). Moreover, Cr was suggested to protect against ischemia [30]. Our results indicated that TCNDS could increase the decreased brain concentration of Cr in MCAO rats to maintain Cr functions. In addition, TCNDS could regulate the disturbed urine concentration of Crn, a product generated from the breakdown of PCr in muscle.

Amino acid metabolism disorders are known to be involved in the cerebral I/R injury [31]. Membrane depolarization leads to the release of the excitatory neurotransmitter Asp after a cerebral I/R injury [32]. Thus, an increased brain level of Asp was observed in MCAO rats. The major inhibitory neurotransmitter GABA has been suggested to suppress excessive neuronal excitability caused by excitatory amino acids [33]. In this study, an elevated level of brain GABA was seen in MACO rats, indicating that the body produced an irritative reaction to the excitability neurotoxicity. Our results indicated that TCNDS could reverse the brain levels of Asp and GABA. Brain levels of NAA, as a neuronal marker, were significantly reduced in MCAO rats, indicating that a cerebral I/R injury caused neuronal damage. In addition, reduced brain levels of NAAG were seen in MCAO rats due to reduction in NAAG synthesis caused by reduced levels of NAA. Our results indicated that TCNDS could reverse the abnormal levels of NAA and NAAG. In addition, TCNDS could regulate the Ace level in brain tissue, possibly related to inhibition in NAA degradation [34].

Inflammation has been reported to be involved in acute ischemic stroke pathogenesis [35], leading to the neuronal cell death after ischemic stroke [36]. In this study, we found that the inflammatory mediator NAG level was elevated in MCAO rats, indicating that a cerebral I/R injury induced inflammation. Our results indicated that TCNDS could reduce the elevated level of urine NAG, exhibiting its potential anti-inflammation property.

## 4. Materials and Methods 

### 4.1. Chemicals and Reagents

NGR1, GSRg1, GSb1, GSRd, HSYA, SI, PA, DZ, VT, and FA were all purchased from Chengdu Mansite Bio-technology Co., Ltd. (Sichuan, China), and the purity of each component was at least 98%, as verified by high-performance liquid chromatography (HPLC, Shimadzu, Kyoto, Japan). Nimodipine tablets were purchased from Bayer Healthcare Company Ltd. (Batch No. BJ29160, Leverkusen, Germany). TTC was purchased from Shanghai Shanpu Chemical Co., Ltd. (Batch No. 20140211, Shanghai, China). Assay kits for LDH, MDA, and SOD were obtained from Nanjing Jiancheng Biological Engineering Institute (Nanjing, China). D_2_O containing 3-(trimethylsilyl)propionic-2,2,3,3-d_4_ acid sodium salt (TSP) was purchased from Teng Long Technology Co., Ltd (Qingdao, China).

### 4.2. Animal and Experiment Design

This study was approved by the Animal Ethics Committee of Guangdong Pharmaceutical University (approval no. gdpulac-2017-018). A total of 80 male Wistar rats (280 ± 20 g) were purchased from the Experimental Animal Center of Guangzhou University of Chinese Medicine (Guangdong, China). All rats were maintained under standard laboratory conditions (a 12 h/12 h light/dark cycle at room temperature of 25 ± 1 °C). After acclimatization for seven days, rats were randomly assigned into five groups, including the sham operation group (as the normal control group), model group (the operation group administrated with normal saline), nimodipine group (as the positive group), L-TCNDS group, and H-TCNDS group.

The control and model groups were given normal saline daily, while the other three groups were administrated with nimodipine (24 mg·kg^−1^·d^−1^), L-TCNDS (158.17 mg·kg^−1^·d^−1^), and H-TCNDS (316.34 mg·kg^−1^·d^−1^), respectively. The proportion of ten components in TCNDS was obtained from NDS active extract by the LC-MS method [14]. Before model establishment, rats in all groups were intragastrically administrated for 4 days. On the night before surgery, all the rats were made to fast but allowed free access to water. On the fifth day, rats were last administrated an hour before surgery.

### 4.3. Animal Model Preparation

The focal cerebral I/R injury was caused by MCAO in rats. Except the normal control group, the MCAO models were prepared in the other four groups. After the rats were anesthetized with 10% chloral hydrate (350 mg/kg), the surgical procedures were performed, consistent with our previous study [13]. Control group rats underwent the same procedures except for the insertion of nylon monofilament. 

### 4.4. Neurobehavioral Dysfunction Evaluation

Neurobehavioral dysfunction of rats was evaluated according to the method previously reported [7,10]. The neurobehavioral dysfunction evaluation was scored on a scale of 0–5. The score criterion was consistent with our previous study [13]. The higher the score, the more serious the neurobehavioral dysfunction. 

### 4.5. Plasma, Urine and Brain Tissue Collection

After 12 h of ischemia reperfusion, 2 mL orbital blood was placed into EP tubes containing heparin sodium, centrifuged at 4 °C (1792× *g*, 10 min), and the supernatant was stored at −80 °C. Before blood collection, urine was obtained within 6–12 h of ischemia reperfusion by using metabolic cages. 50 µL of sodium azide solution (0.1%, *w*/*w*) was added into urine, centrifuged at 4 °C (1792× *g*, 5 min), and the urine supernatant was stored at −80 °C. Finally, all rats were sacrificed by rapid decapitation. The brain tissues were immediately frozen and kept at −20 °C for 15 min, and then five brain slices (2 mm thick) were cut along the coronal plane.

### 4.6. TTC Staining and Measurement of Cerebral Infract Area

The second brain slice was stained with a 2% solution of TTC for 30 min at 37 °C and washed three times with normal saline. The stained brain slice was fixed by placing in 10% formalin solution. After fixation for 24 h, the brain slice was photographed, and then the cerebral infract area was measured by image analysis software (Image-Pro Plus 6.0, Media Cybernetics, Rockville, MD, USA).

### 4.7. HE Staining of Brain Tissue and Pathological Examination

The third brain slice was immediately fixed by placing in 10% formalin solution for 24 h, and embedded in paraffin. 4-µm thick sections were cut and stained with HE in order to observe the pathological changes in brain tissue by light microscopy (×400, Nikon, Japan).

### 4.8. Measurement of LDH, MDA and SOD Levels in Plasma

The plasma samples stored at −80 °C were defrosted at room temperature and then used to measure the plasma levels of LDH, MDA, and SOD, according to the manufacturer’s instructions.

### 4.9. Sample Processing for NMR Measurement

Prior to data acquisition, plasma and urine samples were thawed at room temperature. 130 µL phosphate buffer (0.2 M, Na_2_HPO_4_/NaH_2_PO_4_, pH 7.4), and 100 µL D_2_O were added into a 300 µL plasma sample, centrifuged at 4 °C (1792× *g*, 5 min), and then the supernatant was collected and transferred to a 5 mm NMR tube for NMR measurement. 200 µL phosphate buffer (0.2 M, Na_2_HPO_4_/NaH_2_PO_4_, pH 7.4) and 100 µL D_2_O (1% TSP) were added into 400 µL urine sample, centrifuged at 4 °C (1792× *g*, 5 min), and then the supernatant was collected and transferred to a 5 mm NMR tube for NMR measurement.

About 0.2 g of the remaining three brain slices were weighted. The brain slices were extracted on ice with 3 mL of aqueous acetonitrile solution (50%, *w*/*w*). The extract solution was centrifuged at 4 °C (11,200× *g*, 15 min), and the supernatant was prepared into lyophilized powder by vacuum freeze drying system. Then, the lyophilized powder was dissolved with 400 μL D_2_O (1% TSP), well mixed with 200 µL phosphate buffer (0.2 M, Na_2_HPO_4_/NaH_2_PO_4_, pH 7.4), centrifuged at 4 °C (25,200× *g*, 5 min), and the supernatant was collected and transferred to a 5 mm NMR tube for NMR measurement.

### 4.10. Data Acquisition

All the ^1^H-NMR spectra from plasma, urine, and brain tissue were obtained at 298 K on a Bruker Avance 500 MHz spectrometer (Bruker, Hamburg, Germany). In order to avoid the interference of macromolecules, the one-dimensional Carr-Purcell-Meiboom-Gill (CPMG) pulse sequence with water pre-saturation was adopted to obtain plasma spectrum. A one-dimensional NOESYPR pulse sequence with water suppression was performed to obtain urine and brain tissue spectrum, and the relaxation delay was set to 3 s. 128 transients were acquired into 32 K data points over a spectral width of 10 kHz for plasma and brain tissue spectrum, while 256 transients were collected for urine spectrum. Prior to Fourier transformation, an exponential function with a line-broadening factor of 0.3 Hz was adopted to multiply the free-induction decays (FIDS).

### 4.11. Data Processing and Multivariate Analysis

Each ^1^H-NMR spectrum was phase-adjusted and baseline-corrected manually and then bucketed and automatically integrated using AMIX software (v.3.9.14, Bruker BioSpin, Rheinstetten, Germany). For brain tissue and urine samples, the ^1^H-NMR spectra were referenced to TSP at δ 0.00, while the ^1^H-NMR spectra from plasma were calibrated to lactate (d, δ 1.33). All the ^1^H-NMR spectra were segmented into regions with a width of 0.005 ppm between 0.5 and 9.5 ppm. At the same time, the regions of δ 4.7–5.2 and δ 5.2–6.0 were removed to avoid the interference of water and urea signals, respectively. Prior to pattern recognition analysis, the spectral total integrals normalization method was adopted for every bucketed region in order to compensate for sample concentration differences, and the data set was mean centered.

Both PCA and PLS-DA were performed with the Simca-P^+^ 12.0 software (Umetrics, Sweden). PCA was applied to visualize general clustering among all the groups, and then PLS-DA was applied to enlarge the difference between groups. In addition, one-way analysis of variance was used to the discriminatory variables screened out by PLS-DA, and a P value of less than 0.05 was considered to be statistically significant.

## 5. Conclusions

In summary, this study demonstrated the protective effect of TCNDS against cerebral I/R injuries in rats by using a MCAO model, as evidenced by the fact that TCNDS could improve neurobehavioral dysfunction, reduce cerebral infarct area, and relieve pathological features. In addition, we found that TCNDS could improve the abnormal levels of LDH, MDA, and SOD in plasma induced by a cerebral I/R injury. Furthermore, ^1^H-NMR metabonomic analysis indicated that TCNDS could regulate the disturbed metabolites in plasma, urine, and brain tissue induced by a cerebral I/R injury. The findings of this work revealed that the underlying mechanisms of TCNDS on an ischemic stroke involved multiple pathways, including oxidative stress, energy metabolism, lipid metabolism, amino acid metabolism, and inflammation. Correlation analysis was then performed to further confirm the metabolites involved in oxidative stress. Results of correlation analysis showed that six plasma metabolites had high correlations with plasma LDH, MDA, and SOD. This study provides evidence that NMR-based metabonomics integrated with biochemical assessment can help to better elucidate the underlying mechanisms, as well as the holistic effect of multiple compounds from TCM.

## Figures and Tables

**Table 1:** The results of neurobehavioral performance score and cerebral infarct area ($\bar{x}$ ± s, *n* = 8).

Group	Neurobehavioral Score	Cerebral Infarct Area Percentage (%)
Control group	0.00 ± 0.00	0.00 ± 0.00
Model group	3.75 ± 0.46 ^###^	46.05 ± 6.09 ^###^
Nimodipine group	1.88 ± 0.35 ***	18.44 ± 2.68 ***
H-TCNDS group	2.38 ± 0.52 **	23.93 ± 3.88 ***
L-TCNDS group	2.75 ± 0.46 **	29.53 ± 5.81 **

^###^*p* < 0.001, model group vs control group; ** *p* < 0.01, *** *p* < 0.001, model group vs administration group.

**Table 2:** Effect of TCNDS on plasma lactate dehydrogenase (LDH), malondialdehyde (MDA) and superoxide dismutase (SOD) levels in middle cerebral artery occlusion (MCAO) rats ($\bar{x}$ ± s, *n* = 8).

Group	LDH Level (U·L^−1^)	MDA Level (nmol·mL^−1^)	SOD Level (U·mL^−1^)
Control group	9112.26 ± 1210.75	4.84 ± 0.71	144.68 ± 21.48
Model group	14,963.58 ± 2355.44 ^##^	8.50 ± 1.33 ^###^	78.45 ± 11.23 ^###^
Nimodipine group	10,014.76 ± 1339.41 **	5.56 ± 0.89 **	125.90 ± 20.27 **
H-TCNDS group	10,362.75 ± 1363.21 **	5.88 ± 0.94 **	115.61 ± 17.20 **
L-TCNDS group	11,335.10 ± 1483.57 *	6.44 ± 0.99 *	102.38 ± 14.97 *

^##^*p* < 0.01, ^###^ *p* < 0.001, model group vs control group; * *p* < 0.05, ** *p* < 0.01, model group vs administration group.

**Table 3:** Discriminatory metabolites and their changes in plasma, urine, and brain tissue.

Metabolites	Chemical Shift (ppm)	VIP Score	Model Group	Nimodipine Group	H-TCNDS Group	L-TCNDS Group
Plasma						
VLDL/LDL	0.86m, 1.27m	7.58	↓ ^###^	↑ **	↑ **	↑ *
3-HB	4.15dt, 1.20d	7.18	↓ ^###^	↑ ***	↑ **	↑ *
AcAc	2.28s	5.88	↓ ^###^	↑ ***	↑ **	↑ *
Lac	1.33d, 4.11q	5.58	↑ ^###^	↓ ***	↓ **	↓ *
TMAO/Bet	3.27s	5.06	↑ ^#^	↓ ***	↓ **	↓ *
PUFA	5.30m, 2.77m	3.51	↓ ^###^	↑ ***	↑ **	↑ **
Urine						
Crn	3.05s, 4.06s	5.02	↑ ^###^	↓ ***	↓ **	↓ *
AcAc	2.28s	2.97	↑ ^###^	↓ ***	↓ **	↓ *
Aco	2.23s	2.42	↑ ^###^	↓ ***	↓ **	↓ *
NAG	2.04s	2.24	↑ ^###^	↓ ***	↓ **	↓ *
DMA	2.72s	1.64	↑ ^###^	↓ ***	↓ **	↓ *
3-HB	4.15dt, 1.20d	1.63	↑ ^###^	↓ **	↓ **	↓ *
Brain tissue						
Lac	1.33d, 4.11q	8.25	↑ ^###^	↓ ***	↓ **	↓ *
Cr/PCr	3.04s, 3.93s	6.72	↓ ^###^	↑ **	↑ **	↑ *
Ace	1.92s	6.64	↑ ^###^	↓ ***	↓ **	↓ *
NAA	2.02s, 2.49dd, 4.39dd	5.67	↓ ^###^	↑ **	↑ **	↑ *
m-Ins	3.53dd, 3.63t, 4.07t	5.60	↑ ^###^	↓ ***	↓ ***	↓ **
GABA	2.30t, 1.89m	5.24	↑ ^###^	↓ ***	↓ **	↓ *
Asp	2.81dd, 2.66dd	5.07	↑ ^###^	↓ ***	↓ **	↓ *
Leu	0.96t	4.89	↑ ^###^	↓ ***	↓ ***	↓ **
Ala	1.48d, 3.78q	4.39	↑ ^###^	↓ ***	↓ ***	↓ **
Ile	0.94t	4.32	↑ ^###^	↓ ***	↓ **	↓ *
Cho	3.21s	4.00	↑ ^###^	↓ ***	↓ **	↓ *
NAAG	2.05s	3.52	↓ ^###^	↑ ***	↑ **	↑ *

^#^*p* < 0.05, ^###^ *p* < 0.001, model group vs control group; * *p* < 0.05, ** *p* < 0.01, *** *p* < 0.001, model group vs administration group. ↓ expressed down-regulation, and ↑ expressed up-regulation.

**Table 4:** Pearson correlation coefficients between discriminatory metabolites and biochemical factors for the control group vs the model group in plasma.

Metabolites/Factors	LDH	MDA	SOD
VLDL/LDL	−0.818	−0.851	0.840
3-HB	−0.816	−0.781	0.851
AcAc	−0.752	−0.796	0.766
Lac	0.753	0.815	−0.774
TMAO/Bet	0.746	0.744	−0.843
PUFA	−0.751	−0.791	0.807

## Supplementary Materials

The following are available online, Figure S1: ^1^H-^1^H COSY spectrum of model rat plasma, Figure S2: ^1^H-^13^C HSQC spectrum of model rat plasma, Figure S3: ^1^H-^1^H COSY spectrum of model rat urine, Figure S4: ^1^H-^13^C HSQC spectrum of model rat urine, Figure S5: ^1^H-^1^H COSY spectrum of model rat brain tissue, Figure S6: ^1^H-^13^C HSQC spectrum of model rat brain tissue, Figure S7: PCA scores plots for five groups from plasma (A), urine (B) and brain tissue (C), Figure S8: Plasma PLS-DA validation plots for model group vs control group (A), model group vs nimodipine group (B), model group vs H-TCNDS group (C), and model group vs L-TCNDS group (D), Figure S9: Urine PLS-DA validation plots for model group vs control group (A), model group vs nimodipine group (B), model group vs H-TCNDS group (C), and model group vs L-TCNDS group (D), Figure S10: Brain PLS-DA validation plots for model group vs control group (A), model group vs nimodipine group (B), model group vs H-TCNDS group (C), and model group vs L-TCNDS group (D), Table S1: ^1^H-NMR data and assignments of the metabolites in rat plasma, Table S2: ^1^H-NMR data and assignments of the metabolites in rat urine, Table S3: ^1^H-NMR data and assignments of the metabolites in rat brain tissue, Table S4: The PLS-DA model parameters between model group and other groups from plasma, urine and brain tissue.
