# Supplementary material for: A NMR-Based Metabonomics Approach to Determine Protective Effect of a Combination of Multiple Components Derived from Naodesheng on Ischemic Stroke Rats
Source: Molecules. 2019 May 13;24(9):1831. doi: 10.3390/molecules24091831 (PMC6539225; doi:10.3390/molecules24091831)
Supplement: Supplementary file 1 [file molecules-24-01831-s001.pdf]

Supplementary data

A NMR-Based Metabonomics Approach to Determine Protective Effect of A Combination of Multiple Components Derived from Naodesheng on Ischemic Stroke Rats

Lan Luo [1,2,3], Jiazhen Kang [1,2,3], Qiong He [1,2,3], Yue Qi [1,2,3], Xingyu Chen[1,2,3], Shumei Wang [1,2,3] and Shengwang Liang [1,2,3,*]

[1] School of Traditional Chinese Medicine, Guangdong Pharmaceutical University, Guangzhou 510006, China; luolan1105@126.com (L.L.); KangJZI@163.com (J.K.); m15975633308@163.com (Q.H.); qiyue9339@163.com (Y.Q.); witwick@163.com (X.C.); shmwang@sina.com (S.W.)

[2] Key Laboratory of Digital Quality Evaluation of Chinese Materia Medica of State Administrationof TCM, Guangdong Pharmaceutical University, Guangzhou 510006, China

[3] Engineering & Technology Research Center for Chinese Materia Medica Quality of the Universities of Guangdong Province, Guangdong Pharmaceutical University, Guangzhou 510006, China

* Correspondence: swliang371@126.com; Tel.: +86-020-3935-2172 (S.L.)

Table S1. ^{1}H NMR data and assignments of the metabolites in rat plasma.

Number	Metabolites	Chemical shift(ppm)	Observed
1	VLDL/LDL	0.86m,1.27m	COSY,HSQC
2	isoleucine	0.94t	COSY,HSQC
3	leucine	0.96t	COSY,HSQC
4	valine	0.99d,1.04d	COSY,HSQC
5	3-hydroxybutyrate	4.15dt, 1.20d	COSY,HSQC
6	lactate	1.33d,4.11q	COSY,HSQC
7	alanine	1.48d, 3.78q	COSY,HSQC
8	acetate	1.92s	HSQC
9	N-acetyl glycoprotein	2.04s	HSQC
10	methionine	2.14s,3.77m	COSY,HSQC
11	acetone	2.23s	HSQC
12	Acetoacetate	2.28s	HSQC
13	Pyruvate	2.37s	HSQC
14	glutamine	2.46m	COSY,HSQC
15	creatine /phosphocreatine	3.04s,3.93s	COSY,HSQC
16	TMAO/betaine	3.27s	HSQC
17	glycine	3.56s	HSQC
18	a-glucose	5.24d,3.53m	COSY,HSQC
19	poly unsaturated fatty acid	5.30m,2.77m	COSY,HSQC

Table S2. ^{1}H NMR data and assignments of the metabolites in rat urine.

Number	Metabolites	Chemical shift(ppm)	Observed
1	3-hydroxybutyrate	4.15dt, 1.20d	COSY,HSQC
2	N-acetyl glycoprotein	2.04s	HSQC
3	acetone	2.23s	HSQC
4	acetoacetate	2.28s	HSQC
5	succinate	2.41s	HSQC
6	dimethylamine	2.72s	HSQC
7	dimethylglycine	2.93s	HSQC
8	creatinine	3.05s,4.06s	COSY,HSQC
9	phenylacetylglycine	3.68s	HSQC

Table S3. ^1H NMR data and assignments of the metabolites in rat brain tissue.

Number	Metabolites	Chemical shift(ppm)	Observed
1	isoleucine	0.94t	COSY,HSQC
2	leucine	0.96t	COSY,HSQC
3	valine	0.99d,1.04d	COSY,HSQC
4	lactate	1.33d,4.11q	COSY,HSQC
5	alanine	1.48d, 3.78q	COSY,HSQC
6	acetate	1.92s	HSQC
7	N-acetyl-aspartate	2.02s,2.49dd,4.39dd	COSY,HSQC
8	N-acetylaspartylglutamate	2.05s	HSQC
9	γ-aminobutyric acid	2.30t, 1.89m	COSY,HSQC
10	glutamate	2.36m	COSY,HSQC
11	succinate	2.41s	HSQC
12	glutamine	2.46m	COSY,HSQC
13	aspartate	2.81dd,2.66dd	COSY,HSQC
14	creatine/phosphocreatine	3.04s,3.93s	COSY,HSQC
15	choline	3.21s	COSY,HSQC
16	phosphorylcholine/ glycerophosphocholine	3.23s	COSY,HSQC
17	*scyllo*-inositol	3.35s	HSQC
18	taurine	3.43t	COSY,HSQC
19	*myo*-inositol	3.53dd,3.63t,4.07t	COSY,HSQC
20	glycine	3.56s	HSQC

Table S4. The PLS-DA model parameters between model group and other groups from plasma, urine and brain tissue.

PLS-DA model	A	N	R^2X	R^2Y	Q^2
Plasma					
model group vs control group	2	16	0.642	0.997	0.986
model group vs nimodipine group	2	16	0.534	0.993	0.972
model group vs H-TCNDS group	2	16	0.396	0.998	0.950
model group vs L-TCNDS group	2	16	0.357	0.993	0.897
Urine					
model group vs control group	2	16	0.595	0.998	0.986
model group vs nimodipine group	2	16	0.553	0.998	0.986
model group vs H-TCNDS group	2	16	0.416	0.999	0.979
model group vs L-TCNDS group	2	16	0.389	0.998	0.976
Brain tissue					
model group vs control group	2	16	0.537	0.999	0.983
model group vs nimodipine group	2	16	0.516	0.997	0.971
model group vs H-TCNDS group	2	16	0.408	0.997	0.952
model group vs L-TCNDS group	2	16	0.382	0.996	0.918

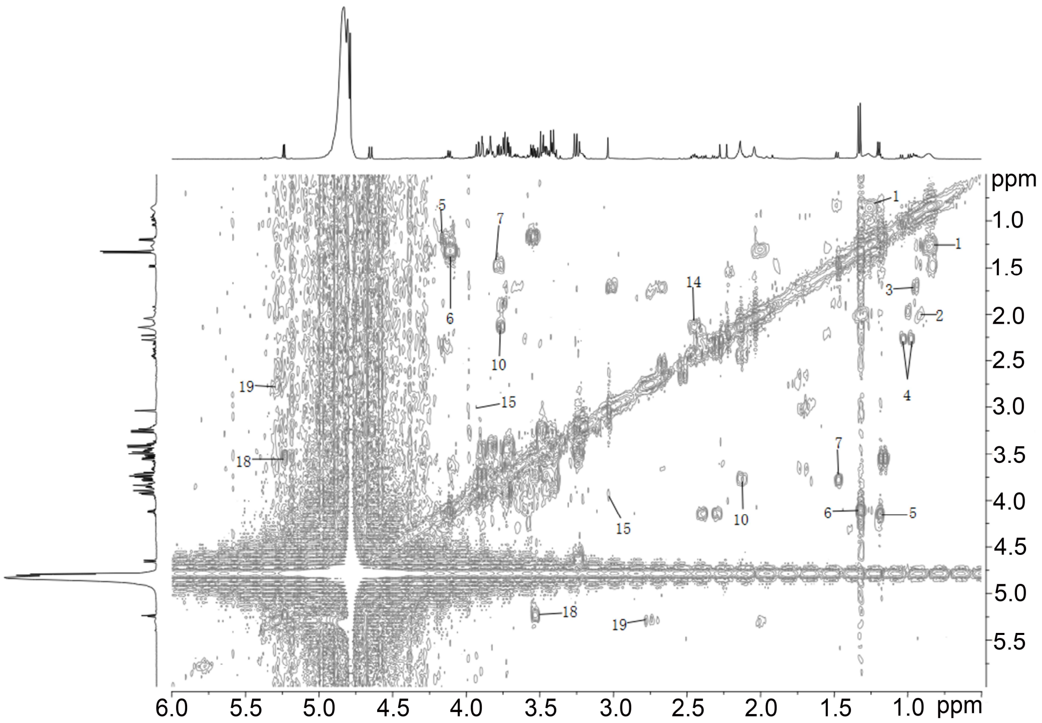

Figure S1. ^{1}H-^{1}H COSY spectrum of model rat plasma

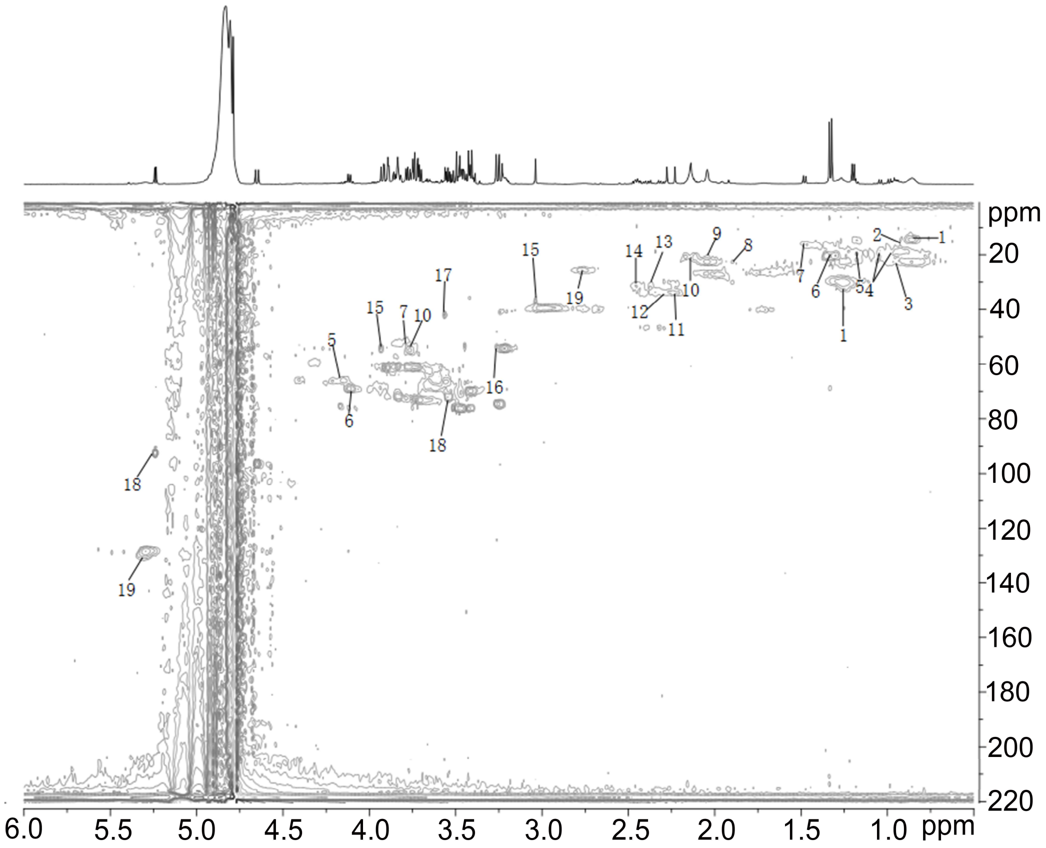

Figure S2. ^{1}H-^{13}C HSQC spectrum of model rat plasma

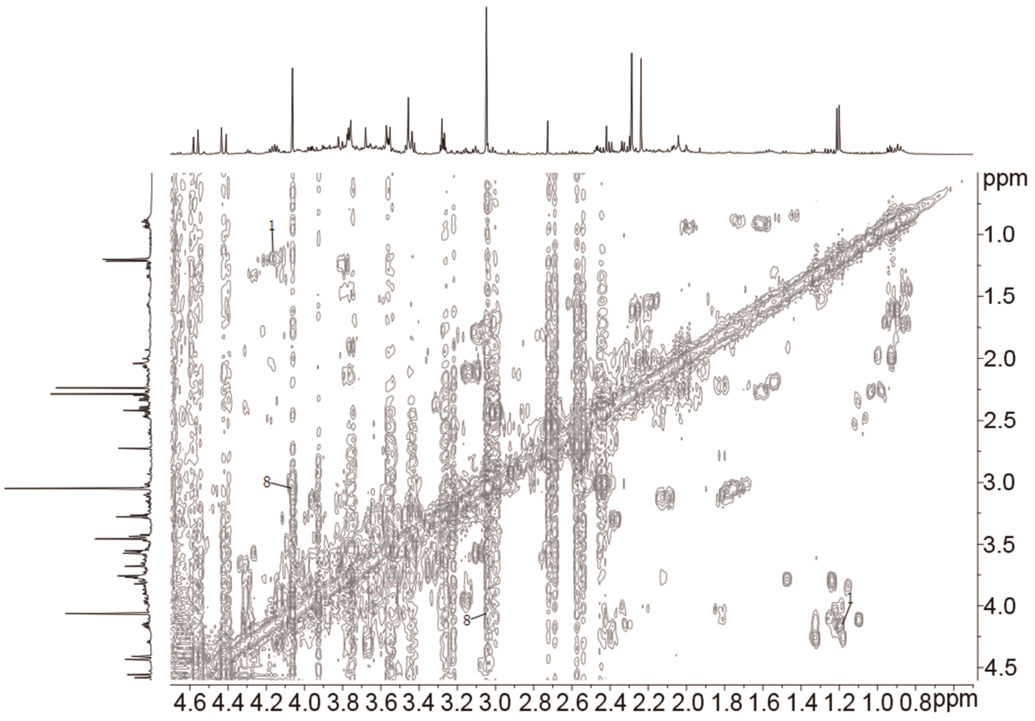

Figure S3. ^1H-^1H COSY spectrum of model rat urine

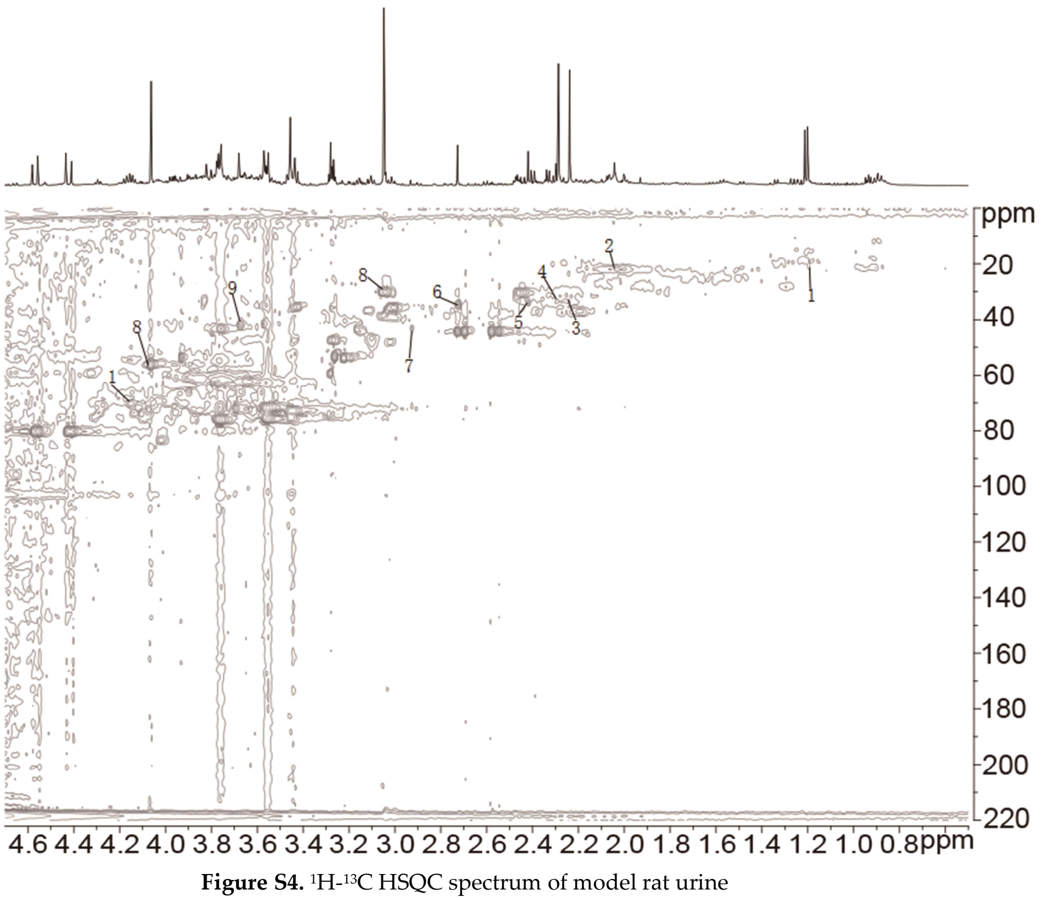

Figure S4. ^1H-^{13}C HSQC spectrum of model rat urine

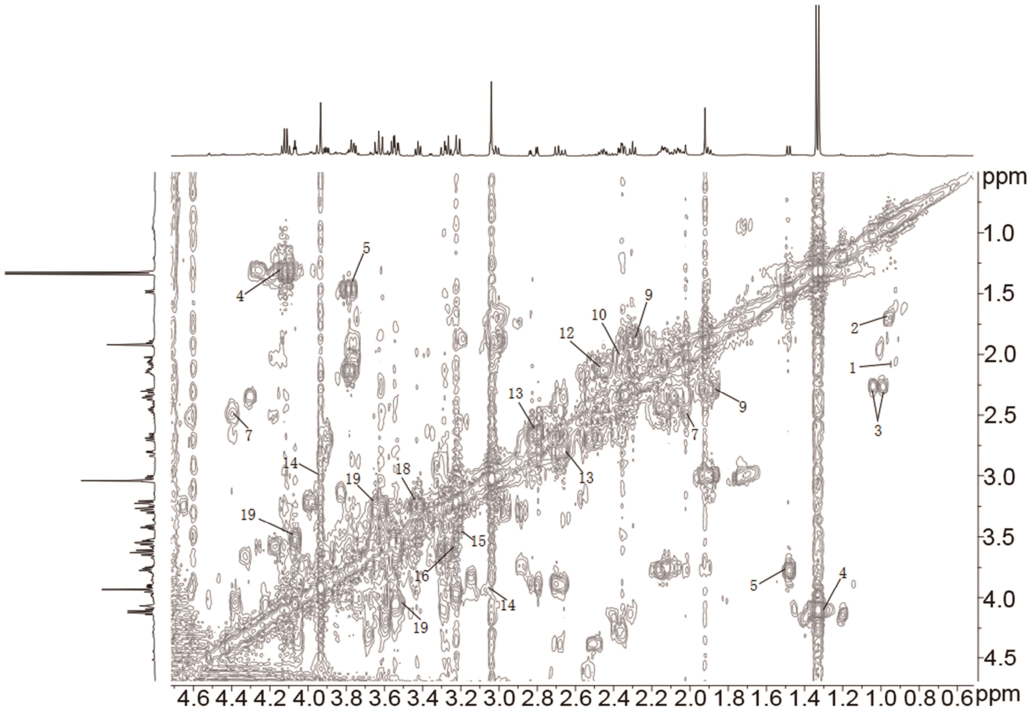

Figure S5. ^{1}H-^{1}H COSY spectrum of model rat brain tissue

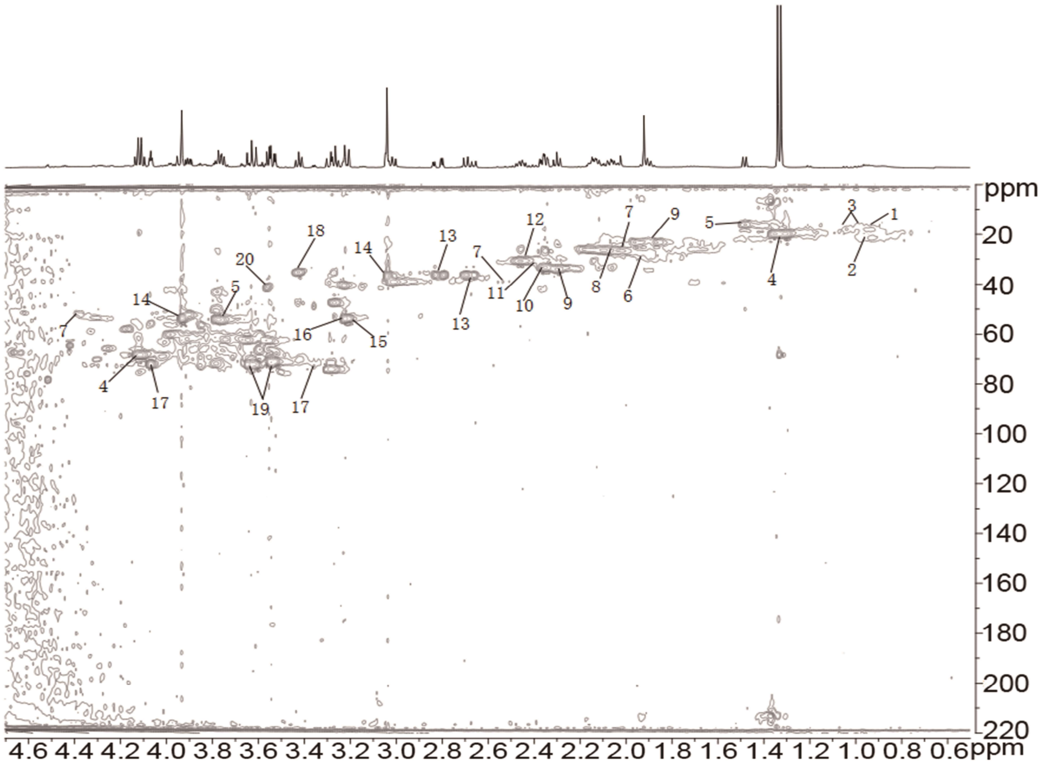

Figure S6. ^{1}H-^{13}C HSQC spectrum of model rat brain tissue

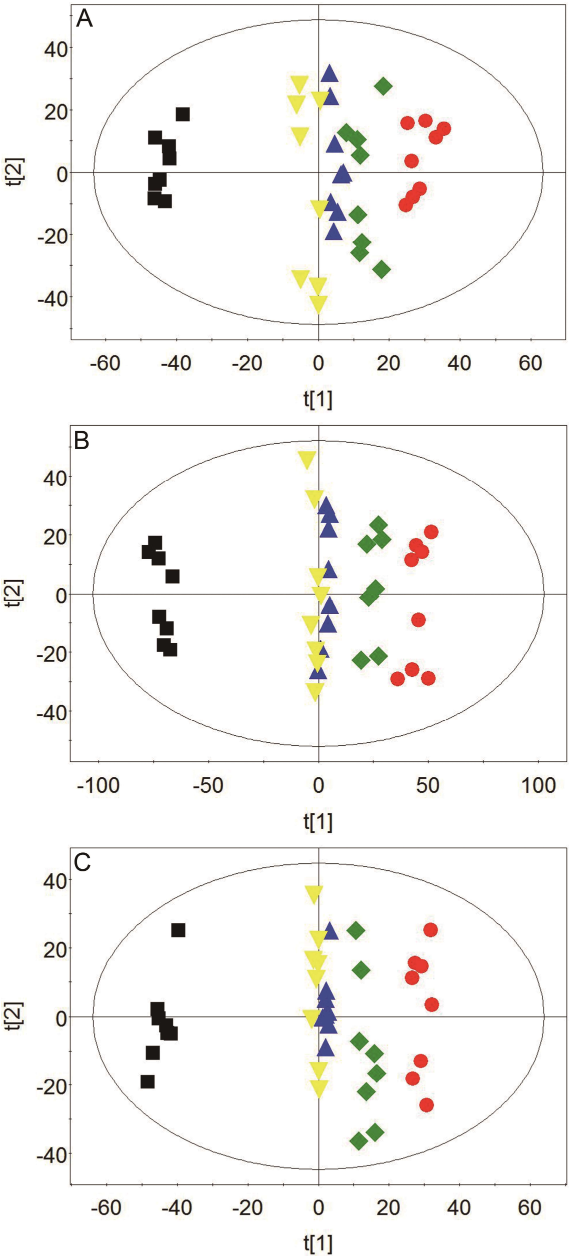

Figure S7. PCA scores plots for five groups from plasma (**A**), urine (**B**) and brain tissue (**C**).
● Control group; ■ Model group; ◆ Nimodipine group; ▲ H-TCNDS group; ▼ L-TCNDS group.

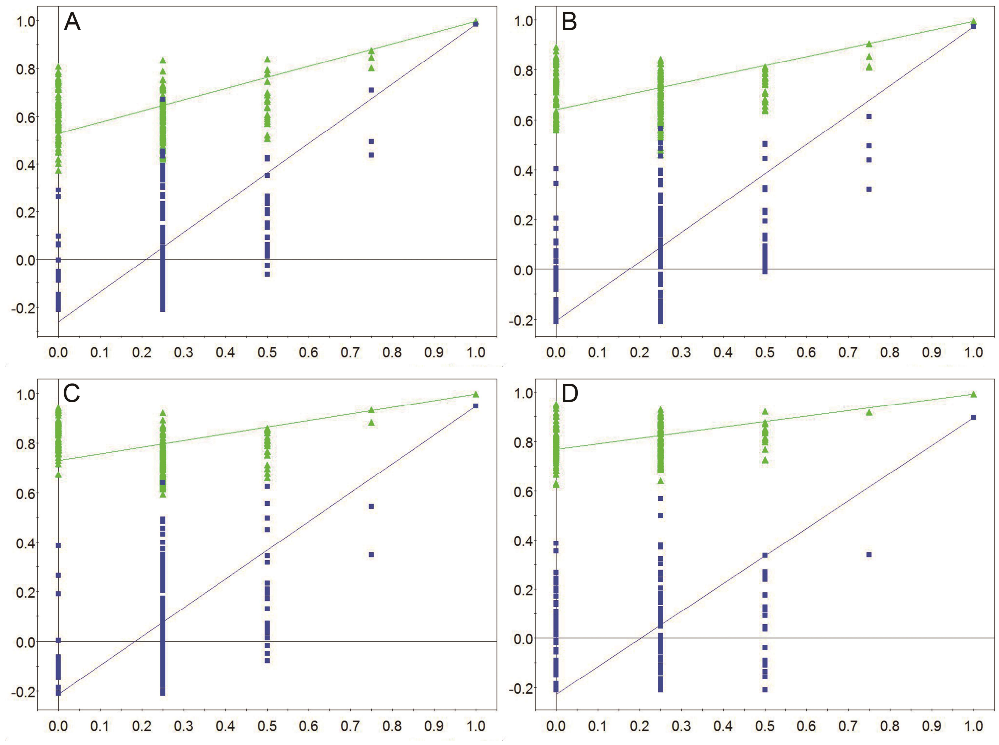

Figure S8. Plasma PLS-DA validation plots for model group vs control group (**A**), model group vs nimodipine group (**B**), model group vs H-TCNDS group (**C**), and model group vs L-TCNDS group (**D**). ▲R2；■Q2

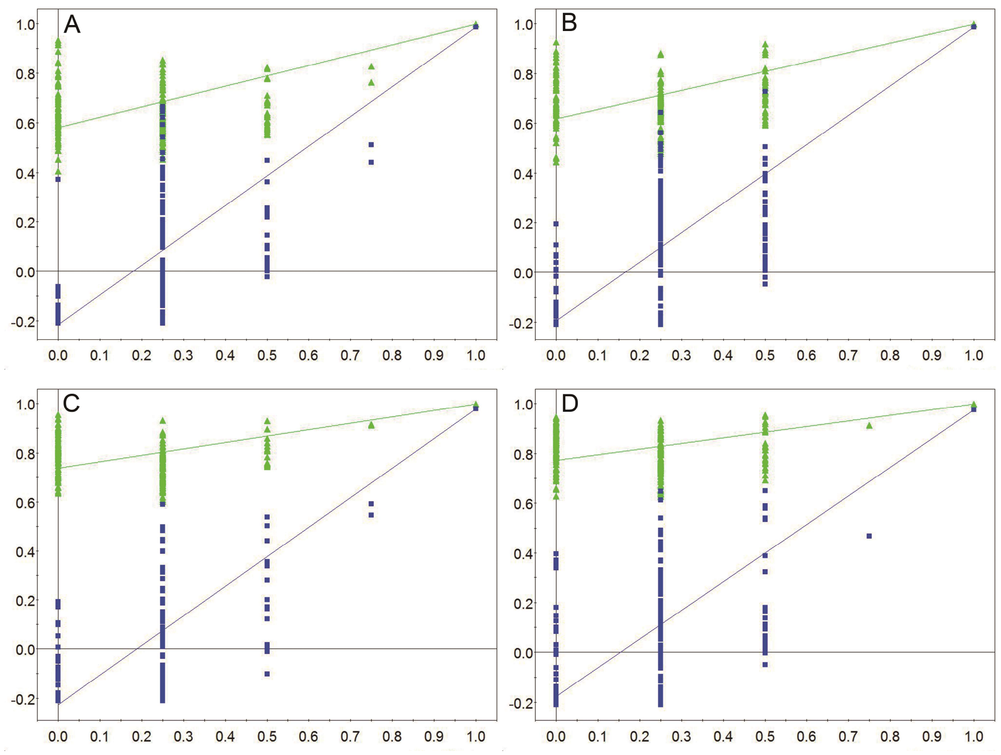

Figure S9. Urine PLS-DA validation plots for model group vs control group (**A**), model group vs nimodipine group (**B**), model group vs H-TCNDS group (**C**), and model group vs L-TCNDS group (**D**). ▲R2；■Q2

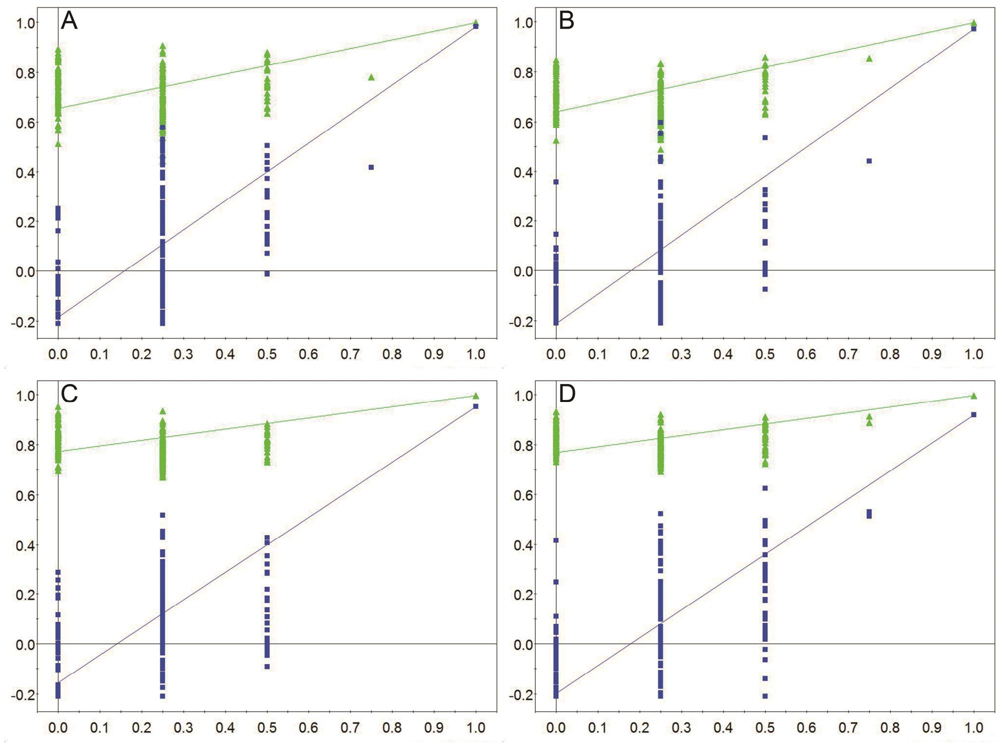

Figure S10. Brain PLS-DA validation plots for model group vs control group (**A**), model group vs nimodipine group (**B**), model group vs H-TCNDS group (**C**), and model group vs L-TCNDS group (**D**). ▲R2；■Q2
